# Birth elicits a conserved neuroendocrine response with implications for perinatal osmoregulation and neuronal cell death

**DOI:** 10.1038/s41598-021-81511-1

**Published:** 2021-01-27

**Authors:** Yarely C. Hoffiz, Alexandra Castillo-Ruiz, Megan A. L. Hall, Taylor A. Hite, Jennifer M. Gray, Carla D. Cisternas, Laura R. Cortes, Andrew J. Jacobs, Nancy G. Forger

**Affiliations:** 1 Neuroscience Institute, Georgia State University, Atlanta, GA 30302 USA; 2 Present Address: Instituto de Investigación Médica M Y M Ferreyra, INIMEC-CONICET-UNC, Córdoba, Argentina

**Keywords:** Neuroscience, Development of the nervous system

## Abstract

Long-standing clinical findings report a dramatic surge of vasopressin in umbilical cord blood of the human neonate, but the neural underpinnings and function(s) of this phenomenon remain obscure. We studied neural activation in perinatal mice and rats, and found that birth triggers activation of the suprachiasmatic, supraoptic, and paraventricular nuclei of the hypothalamus. This was seen whether mice were born vaginally or via Cesarean section (C-section), and when birth *timing* was experimentally manipulated. Neuronal phenotyping showed that the activated neurons were predominantly vasopressinergic, and vasopressin mRNA increased fivefold in the hypothalamus during the 2–3 days before birth. Copeptin, a surrogate marker of vasopressin, was elevated 30-to 50-fold in plasma of perinatal mice, with higher levels after a vaginal than a C-section birth. We also found an acute decrease in plasma osmolality after a vaginal, but not C-section birth, suggesting that the difference in vasopressin release between birth modes is functionally meaningful. When vasopressin was administered centrally to newborns, we found an ~ 50% reduction in neuronal cell death in specific brain areas. Collectively, our results identify a conserved neuroendocrine response to birth that is sensitive to birth mode, and influences peripheral physiology and neurodevelopment.

## Introduction

Birth is an extraordinary event for the placental mammalian fetus, involving major physiological changes in key peripheral organs that are required to survive ex utero^1,2^. The brain houses the control centers of peripheral functions that undergo changes at birth, and the sudden exposure to the many novel stimuli that occur upon delivery requires neural integration and processing. Surprisingly, however, little is known about whether and how birth affects brain activity.

Several studies based on electrophysiological recordings of rodent brain slices in vitro demonstrate perinatal changes in neural activity that may prepare the fetal/newborn brain for birth. In the hippocampus and neocortex, for example, neural network activity is reduced perinatally^3,4^, whereas the newborn piriform cortex and amygdala show spontaneous oscillatory bursting that results from coupled respiratory activity in the brainstem^5^. However, the ability to capture changes in neural activity triggered by birth using this approach is limited, due to the time and ex vivo procedures required to prepare and record from brain slices (i.e., any changes must be preserved through the severing of long-distance projections, slice recovery, and cell patching).

In vivo analyses of brain activity at birth have been performed by examining changes in the expression pattern of the immediate early gene *c-fos* or its protein product^6–8^. This method has the important advantages of allowing for pre- and postnatal measures, as well as simultaneous analyses of many brain regions. Most existing reports, however, either used whole brain homogenates^6^, which cannot address the site(s) of activation, or examined a single time-point^7,9^, which cannot establish whether the activation is actually induced by birth. Thus, the extent to which specific brain nuclei and neural subtypes are selectively activated at delivery or what role they play in the fetus/newborn is unknown.

We therefore examined expression of c-Fos at several time points before and after birth in the rodent brain. Given the dramatic nature of a vaginal birth, we anticipated widespread cell activation. Instead, we found striking activation limited to discrete hypothalamic nuclei, including the suprachiasmatic nucleus (SCN), supraoptic nucleus (SON), and paraventricular nucleus (PVN), which constitute the main sites of production of the neuropeptide vasopressin (VP)^10–12^. Clinical reports spanning four decades have noted that a vaginal birth is accompanied by a massive surge of VP in umbilical cord blood of the human newborn, and that this surge is muted in neonates delivered by Cesarean section (C-section)^13–15^. Nonetheless, what triggers the VP surge, its neuroanatomical basis, and the functional significance of the surge (if any) remain unknown. Here, we examined these questions in mice and rats and report that the transition to *ex utero* life elicits a neuroendocrine response that is conserved across species, sensitive to birth mode, and may have important consequences for peripheral physiology and neurodevelopment.

## Results

### Birth activates discrete brain areas

**Figure 1:**
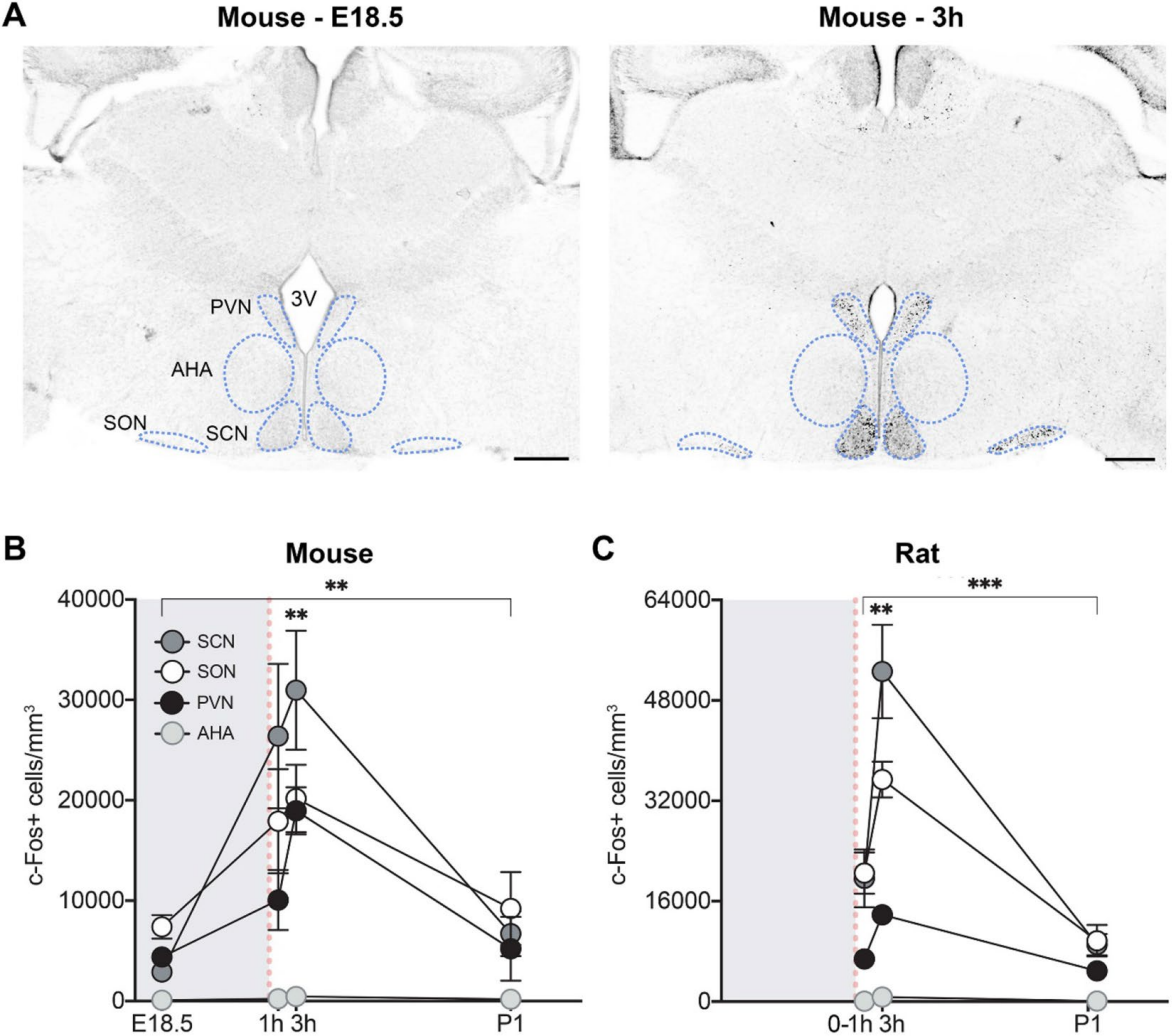
Birth induces neural activation in specific hypothalamic regions of mice and rats. **(A)** Photomicrographs show that in comparison to 12 h before expected delivery (E18.5), there is elevated c-Fos immunoreactivity in the mouse SCN, SON, and PVN at 3 h after birth. 3V, third ventricle. Scale bar: 300 µm. **(B)** Quantification shows a significant main effect of age on c-Fos+ cell density in the SCN, SON, PVN, and AHA (asterisks over bracket, **P < 0.005), with neural activation peaking at 3 h post-delivery in all regions [**P < 0.004 compared to E18.5 and postnatal day (P) 1]. However, peak density of labeled cells in the AHA was less than one-fortieth of that in the other regions. (**C**) Similar effects of birth on c-Fos immunoreactivity were observed in the rat SCN, SON, PVN, and AHA [asterisk over bracket corresponds to main effect of age (***P < 0.0009 for all brain areas; post hocs: **P < 0.004 for 3 h compared to 0-1 h and P1)]. Gray shading indicates in utero timepoints; red dotted lines indicate the time of birth. Data are mean ± SEM. n = 10–12 animals per group.

We initially cast a wide net by analyzing c-Fos immunoreactivity throughout the perinatal mouse forebrain, midbrain, and rostral hindbrain. Because the average gestation length in C57BL/6J mice is 19.3 days^16^, we examined brains on embryonic day (E)18.5 (the day before expected birth), and at 1 h, 3 h, and one day postnatal (P1; specifically, 27 h) after a vaginal delivery. We found a striking pattern of activation in several hypothalamic nuclei, including the SCN, SON, and PVN. In all three regions, the density of c-Fos+ cells was low at E18.5, high at 3 h postnatal, and returned to baseline levels at P1 (main effect of age: Kruskal–Wallis H_3_ > 13.28, *P* < 0.004 in each case) (Fig. 1A,B and Fig. S1). By contrast, c-Fos+ cell density in neighboring hypothalamic regions, such as the anterior hypothalamic area (AHA), was extremely low at all timepoints (Fig. 1A,B). The perinatal differences in c-Fos+ cell density were not due to circadian effects because pups that were born at very different times of the day had similar high c-Fos labeling at 3 h postnatal (Fig. S2). Moreover, c-Fos was high at 3 h but low at P1 (27 h after birth), despite the fact that pups in the P1 group were collected at the same time of day as their littermates in the 3 h group.

Other brain regions with visibly increased neural activation at 3 h postnatal included the caudate-putamen, lateral habenula, paraventricular thalamus, subfornical organ, piriform cortex, medial amygdala, dorsal hypothalamus, periaqueductal gray, median raphe nucleus, and locus coeruleus (Fig. S3). We quantified c-Fos+ cell density in several of these areas at E18.5, 1 h, 3 h, and P1, and confirmed a main effect of age (H_3_/F_3,41–43_ > 12.17, *P* < 0.005 in each case): c-Fos+ cell density was again low at E18.5, high at 3 h postnatal, and returned to baseline levels at P1 (Fig. S3). However, with the exception of the subfornical organ, c-Fos+ cell density at 3 h was not as high as in the SCN, SON, and PVN.

To determine whether the neural activation at birth generalized to other species, we analyzed c-Fos+ cell density in the rat hypothalamus within 1 h of birth (0-1 h), and at 3 h and P1. Because expression of c-Fos protein generally peaks 1-3 h after stimulus onset^17,18^, the 0-1 h time point likely reflected activity while still in utero. We found a very similar pattern of neural activation, with peak levels of c-Fos+ cells in the SCN, SON, and PVN at 3 h (main effect of age in each case, F_2,32_ > 18.28, *P* < 0.0001) and very little activation in most other regions (Fig. 1C). However, in contrast to the mouse brain, we also found sex differences in c-Fos+ cell density: c-Fos+ cell density in the SCN was greater in females than in males 0-1 h after birth, whereas c-Fos+ cell density was greater in males at P1 in the SCN and SON (Fig. S4).

### Birth activates VP and OT neurons

The brain regions with the greatest activation at 3 h postnatal (SCN, SON, and PVN) all house prominent groups of neuropeptide-containing neurons. VP is the main output peptide of the SCN^19^, and the SON is comprised primarily of magnocellular VP and oxytocin (OT) neurons that project to the posterior pituitary and release their contents directly into the bloodstream^20^. The PVN contains (i) magnocellular VP and OT neurons that project to the posterior pituitary or to other brain regions via axon collaterals; (ii) parvocellular VP, OT, and corticotropin releasing hormone (CRH) neurons that project to the median eminence as well as to other brain regions; and (iii) neurons containing many other peptides^21–24^.

The pattern of neural activation at birth was intriguing because long-standing clinical reports show that human newborns experience extremely high circulating levels of VP^25,26^. In addition, VP and OT have been implicated in protecting the neonatal rodent brain from the stress of birth by decreasing perinatal neural activity^3,4^, and CRH neurons in the PVN are activated around the time of birth in sheep^27^. Therefore, in a new cohort of mice we phenotyped the activated neurons in the perinatal SCN, SON, and PVN, focusing on VP, OT, and CRH. Double-label immunofluorescence was used to examine co-labeling of c-Fos with VP and OT, and c-Fos immunofluorescence was performed in a CRH-tdTomato reporter mouse.

**Figure 2:**
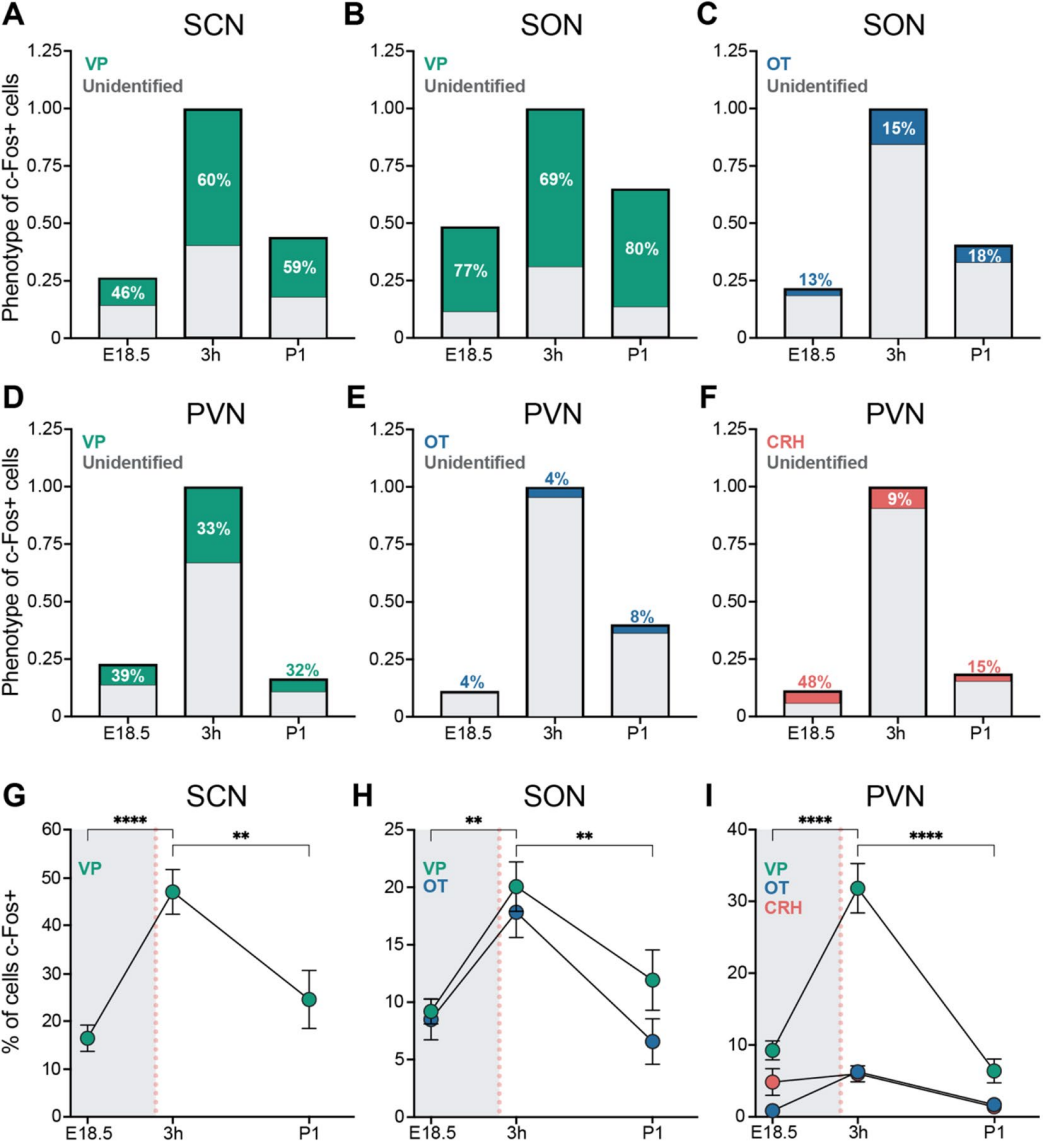
Phenotyping of activated cells in the SCN, SON, and PVN in perinatal mice. **(A–F)** Percent of c-Fos+ cells at E18.5, 3 h postnatal and P1 that co-label for VP, OT, or CRH. The height of each bar reflects the c-Fos+ cell density at each age relative to 3 h. c-Fos+ cell density was higher at 3 h compared to E18.5 or P1 in all regions. (**A**) In the SCN, the majority of c-Fos+ cells at 3 h and P1 were VP + (green) and a smaller percentage were unidentified (gray). In the SON, the majority of c-Fos+ cells were VP + at all timepoints **(B)**, and a smaller percentage were OT + (blue) **(C)**. In the PVN, one third of all c-Fos+ cells were VP + **(D)**, and a smaller percentage were OT + **(E)** or CRH + (red) **(F)** at 3 h; about half of the c-Fos+ cells were unaccounted for. **(G–I)** The percent of VP, OT, and CRH cells at each timepoint that were c-Fos+. In the SCN **(G)**, SON **(H)** and PVN **(I)**, significantly more VP neurons were c-Fos+ at 3 h postnatal than at E18.5 or P1. In the SON, OT neurons also had increased c-Fos labeling at 3 h compared to E18.5 and P1. Gray shading indicates in utero timepoints; red dotted line indicates the time of birth. Data are mean ± SEM. N at each age = 11–12 for VP and OT, and 6–8 for CRH. **P < 0.005, ****P < 0.0001.

**Figure 3:**
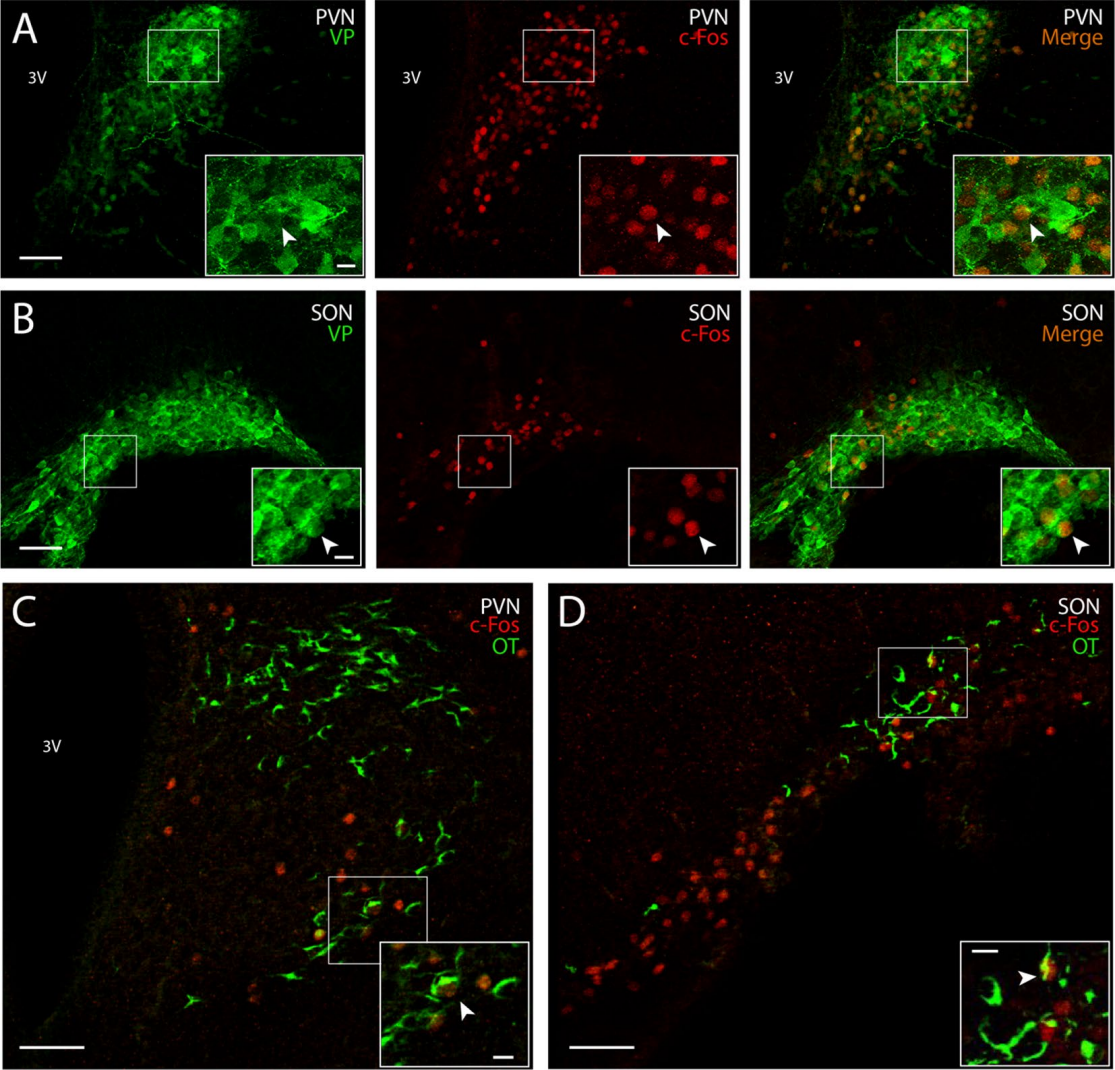
Dual label immunofluorescence of c-Fos+ with vasopressin or oxytocin. In the PVN (**A**) and SON (**B**), many c-Fos+ cells (red, nuclear stain) are positive for vasopressin (green, cytoplasmic stain). A smaller number of c-Fos+ cells in the PVN (**C**) and SON (**D**) co-label for oxytocin. All images are from pups collected 3 h after birth. Arrows point to double-labeled cells. 3V, third ventricle. Scale bar: 50 µm (main images); 10 µm (insets).

We again saw peak c-Fos+ cell number in the SCN, SON, and PVN at 3 h (main effect of age in each case, H_2_/F_2,32_ > 6.709, *P* < 0.004), although the overall density of c-Fos+ cells was lower in this experiment than in Fig. 1, likely because immunofluorescence is less sensitive than 3,3′-diaminobenzidine labeling. Interestingly, the majority of all c-Fos+ neurons were vasopressinergic in the SCN at 3 h and P1, and in the SON at all ages (Figs. 2A–C, 3). In the much more heterogeneous PVN, VP neurons accounted for at least one third of all c-Fos+ cells (Figs. 2D–F; 3). OT+ and CRH+ neurons, by contrast, made up a relatively small percentage of all c-Fos+ cells.

We also performed the reverse analysis, calculating the percent of VP+, OT+ or CRH+ neurons that were c-Fos+ in each nucleus (the total number of neurons of each phenotype at each age is presented in Table S1). In the SCN, which does not contain OT or CRH neurons, almost half of all VP neurons showed c-Fos labeling at 3 h post-delivery (main effect of age: F_2,32_ = 10.94, *P* = 0.0002; E18.5 vs. 3 h, *P* < 0.0001; 3 h vs. P1, *P* = 0.002) (Fig. 2G). In the SON, 20% of VP and 18% of OT neurons were c-Fos+ at 3 h post-delivery (main effect of age: F_2,32_ = 13.47, *P* < 0.0001) (Fig. 2H). In the PVN, a third of all VP neurons were c-Fos+ at 3 h, which was significantly greater than at E18.5 or P1 (F_2,32_ = 42.75, *P* < 0.0001 for main effect of age; *P* < 0.0001 in post hoc tests) (Figs. 2I, 3). Only 6% of OT + and 6% of CRH + neurons in the PVN were c-Fos+ at 3 h, although this was a significant increase from E18.5 (< 1%) for OT (*P* < 0.04).

### Birth activates VP neurons regardless of delivery mode

Given the lag between stimulus onset and c-Fos protein accumulation^17,18^, the activation we observed at 3 h postnatal was presumably triggered by stimuli associated with birth or the immediate peripartum period. A vaginal birth is a complex stimulus involving marked hormonal changes associated with labor, mechanical stimuli associated with uterine contractions, passage through the birth canal, and exposure to the *ex utero* environment^28^. All of these factors, with the exception of exposure to the *ex utero* environment, are altered following a C-section delivery. We took advantage of the inherent differences between a vaginal and a C-section birth to determine whether specific aspects of birth are required to trigger neural activation in the hypothalamus.

**Figure 4:**
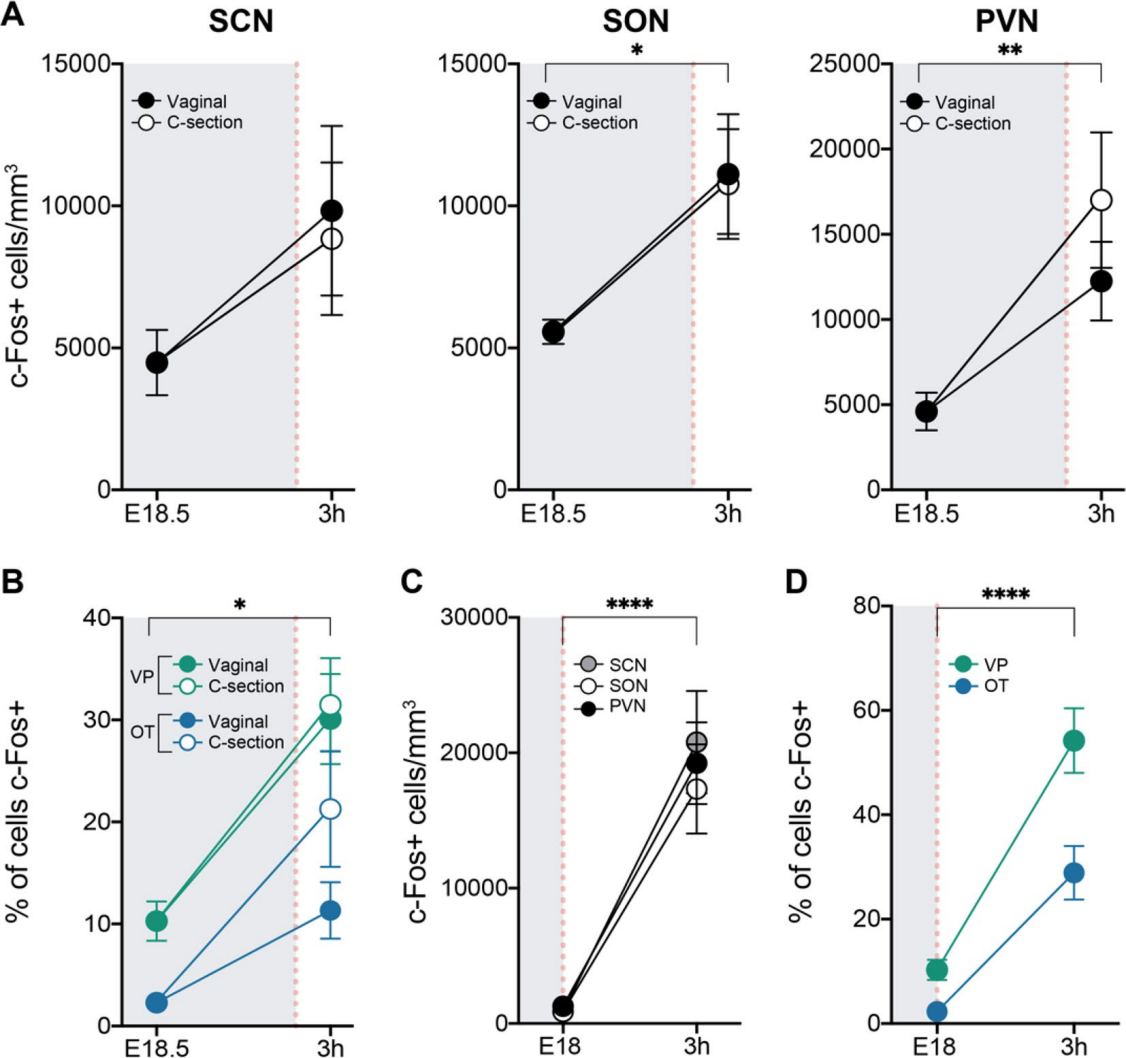
Neural activation is independent of birth mode or birth timing. (**A**) c-Fos+ cell density in pups born vaginally or by C-section. Neural activation was higher at 3 h postnatal compared to E18.5 in the SCN, SON, and PVN, although this was significant only for the PVN and SON. In all three brain areas, the neural activation at 3 h postnatal was similar whether pups were delivered vaginally (black circles) or by C-section (open circles). **(B)** Phenotyping in the PVN showed that both VP (green) and OT (blue) neurons were activated at 3 h post-delivery compared to E18.5 (asterisk over bracket; *P* < 0.0001 for VP and *P* = 0.02 for OT), and this activation was similar whether pups were delivered vaginally or by C-section. (**C**) c-Fos+ cell density after an early birth. As was seen for on-time deliveries, the density of c-Fos+ cells was elevated at 3 h postnatal in the SCN, SON, and PVN in pups delivered one day early (*P* < 0.0001 in all cases). **(D)** Both VP and OT neurons in the PVN were activated 3 h after an early delivery (asterisks over bracket, ****P < 0.0001 in both cases), and the effect was greater for VP neurons (*P* < 0.0001). Gray shading indicates in utero timepoints; red dotted line indicates the timing of birth. Data are mean ± SEM. n = 11–13 animals per group.

Timed-pregnant mouse dams were checked hourly for births starting on E18.5. C-section deliveries were yoked to vaginal births to eliminate possible confounds of gestation length and time of day, as previously^29^, and all offspring (whether born vaginally or by C-section) were placed on a warming pad immediately after birth to control for absence of the dam following a C-section. The activation did not differ significantly whether pups were delivered vaginally or by C-section (Fig. 4A).

In addition, VP neurons were activated regardless of birth mode. We analyzed c-Fos/VP, or c-Fos/OT co-labeling in the PVN 3 h after a vaginal or C-section birth. There was greater activation of VP than of OT neurons in the vaginal group (*P* < 0.0001), and the percent of c-Fos/VP neurons was nearly identical after a C-section delivery (Fig. 4B). Similarly, while activation of OT neurons after C-section delivery appeared somewhat higher than after a vaginal delivery, the difference was not significant.

### Neural activation at 3 h postnatal is independent of birth timing

It remained possible that the increase in c-Fos labeling at 3 h postpartum was not related to birth at all, but occurred instead due to a developmentally-programmed induction at 19 days post-conception. To test this, we examined the neural activation in animals that were delivered one day early (E18). We reasoned that if birth triggers the neural activation, then c-Fos+ cell density should increase 3 h after an early birth, even though labeling in utero on E18 is normally low (Fig. 1). Indeed, we observed robust neural activation at 3 h postnatal in pups delivered on E18 in the SCN, SON, and PVN (*P* < 0.0001 in all cases) (Fig. 4C). In fact, the fold-induction was greater 3 h after birth on E18 than after an on-time delivery (E19) (compare Figs. 4 and 1). Moreover, phenotyping of PVN cells after an early birth again showed activation of both VP and OT neurons, with greater activation of VP neurons, particularly at 3 h [Fig. 4D; main effects of age (F_1,22_ = 58.44, *P* < 0.0001), neural phenotype (F_1,22_ = 20.81, *P* = 0.0002), and an age-by-neural phenotype interaction (F_1,22_ = 5.617, *P* < 0.03) in a two-way ANOVA].

### Copeptin levels are markedly elevated in perinatal mouse plasma

Copeptin is widely used as a surrogate marker of VP because it is derived from the same precursor molecule, is released into systemic circulation in equimolar amounts, and is a larger and more stable peptide^30,31^. As mentioned above, human neonates experience extremely high circulating levels of copeptin/VP, and levels are higher after a vaginal birth than after a C-section delivery^13–15^. To determine whether birth is associated with a peripheral release of VP in the mouse, we measured plasma levels of copeptin at three perinatal timepoints (E18.5, and 0 h and 3 h after birth), and compared them to levels in pregnant or non-pregnant adults. We also evaluated the effect of birth mode on peripheral VP release in the mouse by collecting plasma 0 h and 3 h after a C-section delivery.

**Figure 5:**
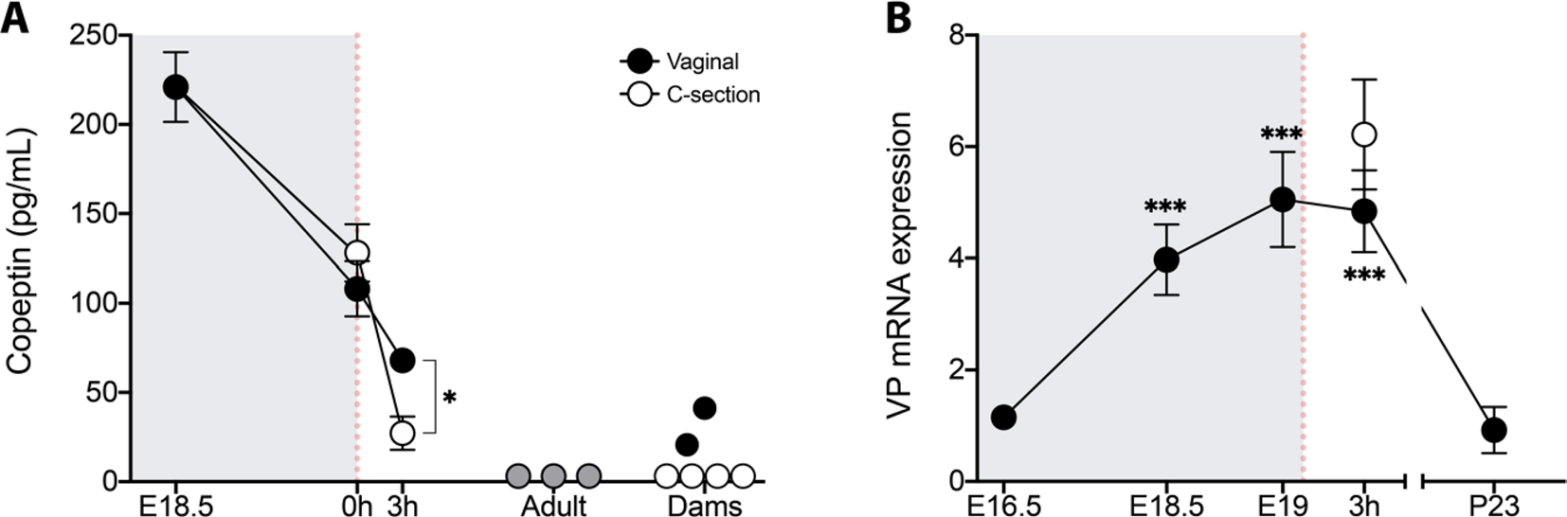
Plasma copeptin and neural VP expression are elevated perinatally. (**A**) Plasma copeptin levels were extremely high in fetuses on E18.5. Levels dropped somewhat at birth (0 h) and further by 3 h postnatal. Copeptin levels decreased more abruptly in pups delivered by C-section than in those delivered vaginally, and differed significantly between the two birth-mode groups at 3 h (*P < 0.04). Copeptin was undetectable in non-pregnant adults (gray circles) and in all pregnant dams that delivered by C-section (open circles). Dams that delivered vaginally (black circles) had detectable copeptin levels that, nonetheless, were significantly lower than their pups at 0 h (*P* < 0.04). Number of samples per group: E18.5 = 6; 0 h vaginal = 4; 0 h C-section = 4; 3 h vaginal = 4; 3 h C-section = 4; non-pregnant adults = 3; dams = 6. (**B**) VP mRNA expression in the hypothalamus increases five-fold in the last few days of gestation. VP mRNA was low three days before expected delivery (E16.5), but increased markedly 12 h prior to birth (E18.5). VP expression remained elevated at E19 and 3 h postnatal, and dropped to prenatal levels by weaning (P23). ***P < 0.0003 vs. E16.5 and P23. Birth mode had no effect on VP expression at 3 h. Gray shading indicates in utero timepoints; red dotted line indicates the timing of birth. Data are mean ± SEM. n = 8–12 animals per group.

Plasma copeptin was below the detection limit of the assay (4.36 pg/mL) in all non-pregnant adults. By contrast, levels ranged from 21.75 to 287.7 pg/mL in all fetuses and vaginally-born neonates (Fig. 5A). Therefore, we conservatively estimate a ≥ 50-fold elevation of copeptin in E18.5 fetuses and up to a 30-fold elevation in neonates compared to adults.

Using two-way ANOVA to compare copeptin levels between the vaginal and C-section groups at 0 h and 3 h, we found a significant main effect of age (F_1,12_ = 33.17, *P* < 0.0001), with higher levels at 0 h, and an age-by-birth mode interaction (F_1,12_ = 6.16, *P* < 0.03). Copeptin levels were similar between groups at 0 h, but were lower in the C-section group than the vaginal group at 3 h (*P* < 0.04).

To test the prediction that copeptin is of fetal and not maternal origin, we compared levels in neonates to those of their dams. As was seen for non-pregnant adults, all but two dams had undetectable levels of copeptin. In the dams with detectable copeptin, levels were significantly lower than in their pups (delivered vaginally) at the same timepoint (0 h, *P* < 0.04) (Fig. 5A). Thus, our copeptin data suggest that: (1) perinatal mice have very high levels of circulating VP, (2) VP is of fetal/neonatal origin, and (3) VP levels in newborns are higher following a vaginal delivery than after C-section.

### Rapid increase in vasopressin production just prior to birth

To better understand the underpinnings of the VP surge at birth, we examined VP mRNA perinatally (E16.5, E18.5, E19 and 3 h postnatal) and at P23 in hypothalamic brain punches that included the PVN. We found a striking, 500% increase in VP gene expression between E16.5 and E18.5/E19 that dropped back down to prenatal levels by weaning (Fig. 5B). Expression was equally high at 3 h postnatal regardless of birth mode, suggesting that the lower copeptin/VP in circulation after a C-section delivery is not due to less production, but instead reflects less release or increased metabolism of copeptin/VP.

### Decreased plasma osmolality after a vaginal delivery

**Figure 6:**
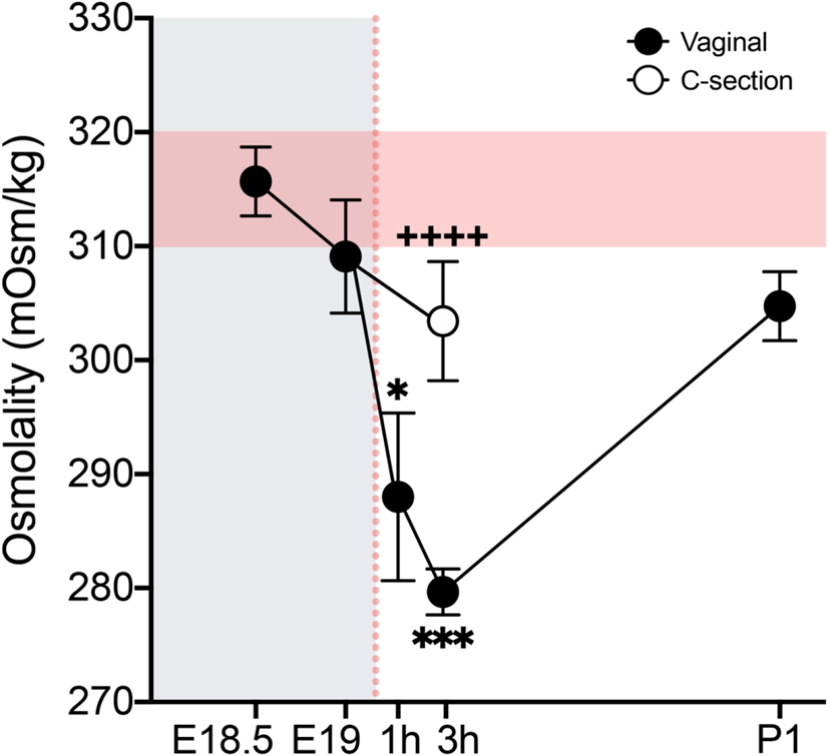
Plasma osmolality is acutely decreased after a vaginal, but not Cesarean, delivery. Relative to prenatal levels or levels at P1, plasma osmolality was decreased at 1 h (*P < 0.05) and 3 h (***P < 0.0002) after vaginal birth. This decrease was not observed in C-section-delivered pups, which had significantly higher plasma osmolality at 3 h postnatal than did vaginally-delivered pups (^++++^*P* < 0.0001). Red horizontal shading indicates normal osmolality range in mouse plasma. Gray shading indicates in utero timepoints; red dotted line indicates the timing of birth. Data are mean ± SEM. n = 6–12 per group.

The function of the copeptin/VP surge in neonates is unknown. One of the most important roles of peripheral VP is to control solute concentration (osmolality) in the blood by increasing water reabsorption from the kidneys^32,33^. We therefore asked whether birth is associated with acute changes in plasma osmolality, and whether this differs by birth mode. To test this, we collected plasma from offspring prenatally (E18.5 and E19), and at 1 h, 3 h, and P1 after a vaginal delivery, as well as 3 h after a C-section delivery. Osmolality of fetuses (E18.5 and E19) was in the expected normal range^34,35^ (Fig. 6). Interestingly, vaginal birth was associated with an acute decrease in plasma osmolality (H_4_ = 33.61, *P* < 0.0001 for overall effect of age; E18.5/E19 vs. 1 h, *P* < 0.05; E18.5/E19 vs. 3 h, *P* < 0.0002) which normalized by P1 (3 h vs. P1, *P* = 0.0003) (Fig. 6). The decrease was not observed in C-section-delivered pups, which had higher plasma osmolality than vaginally-delivered pups at 3 h (*P* < 0.0001). This suggests that the high circulating copeptin/VP after a vaginal birth may transiently boost water retention, thereby decreasing osmolality. Moreover, the difference in copeptin/VP release between vaginal and C-section delivery (Fig. 5A) appears to be of functional significance.

### Vasopressin treatment at birth reduces neuronal cell death in the PVN and AHA

Plasma copeptin/VP levels correlate closely with levels in cerebrospinal fluid in human newborns^36,37^, indicating that birth is associated with concomitant peripheral and central VP release. In rodents, the SCN and PVN are the main source of VP in cerebrospinal fluid^38^, and these regions were highly activated at birth (Fig. 1). Thus, it is likely that VP is centrally released at birth in mice, and VP may play a previously unappreciated role in neonatal brain development.

**Figure 7:**
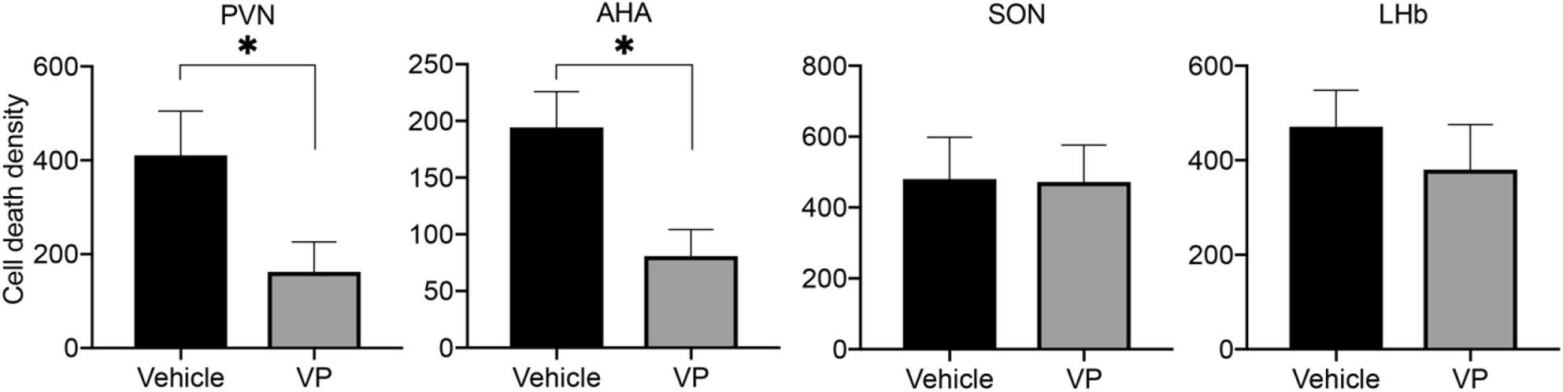
Intracerebroventricular administration of VP at birth reduces neuronal cell death in the PVN and AHA three hours after C-section delivery. Cell death density (activated caspase-3 + cells per mm^3^) was not affected in the SON or LHb of the same animals. Data are mean ± SEM. n = 6 for vehicle and 7 for VP group. *P < 0.05.

VP can be neuroprotective in vitro^39^. We therefore tested the hypothesis that vasopressin protects neurons from developmental neuronal cell death, which is concentrated around the time of birth in mice^40,41^. In several brain regions, neuronal cell death is transiently higher in pups delivered by C-section than in those delivered vaginally^29^. We hypothesized that low copeptin/VP in pups 3 h after a C-section birth may account for the high cell death after C-section. To test this, we injected VP or vehicle into the lateral ventricles of C-section-delivered mice immediately after birth. Brains were collected 3 h later, and the number of cells positive for the cell death marker activated caspase-3 was counted in three brain areas where we previously saw an effect of birth mode on cell death: the PVN, AHA and LHb^29^, as well as in the SON, where we have recently confirmed a similar birth mode effect (unpublished observations). We found that intracerebroventricular injections of VP reduced cell death by over 50% in the PVN (*P* < 0.04) and the AHA (*P* < 0.03), but had no effect in the SON or the LHb (Fig. 7). Interestingly, the cell death densities in the PVN and AHA of vasopressin-treated neonates (162.2 ± 64.02 and 80.86 ± 23.34, respectively) were similar to, or even lower than, those reported previously after a vaginal delivery (265.90 ± 71.34 and 80.07 ± 22.47, respectively)^29^.

## Discussion

We find that birth triggers neural activation in discrete regions of the newborn brain, with prominent activation in hypothalamic nuclei that produce VP. Phenotyping showed that indeed, many c-Fos+ cells were vasopressinergic. Labor and a vaginal delivery were not required to induce neural activation at birth, because c-Fos+ cell density increased at 3 h postnatal whether pups were delivered on E18 or E19, and vaginally or by C-section. Moreover, time of day did not play a role, and we ruled out the possibility that neural activation is triggered by an autonomous developmental program by showing a similar pattern of activation following an early birth. Collectively, these results suggest that activation of hypothalamic VP (and, to a lesser extent OT) neurons is triggered by the transition to life *ex utero*, independent of birth mode, time of day, or gestation length.

One limitation of the current findings is that c-Fos expression does not invariably match other techniques measuring neural activity^42,43^, and is not induced by all types of stimuli^44–46^. Nonetheless, its cellular resolution and low basal expression, as well as the possibility to perform brain-wide mapping of activity in utero and ex utero, make c-Fos a powerful tool for the study of neural activation patterns in response to birth. Our observation that prominent activation of VP neurons was associated with elevated peripheral vasopressin further supports the notion that c-Fos reflected neural activity.

Human and sheep fetuses show extremely high copeptin/VP levels during labor and at parturition^25,47^. Our finding that VP neurons in the SON and PVN are activated at birth provides a neuroanatomical correlate for these long-standing observations. In addition, we found a remarkable ramping up of hypothalamic VP mRNA expression in the 2–3 days before birth, and much higher expression at birth than three weeks later. This pattern is in accord with a previous report on the ontogeny of the vasopressin system in mice^48^. Because synthesis and release of VP are not necessarily coupled processes^49–51^, it is possible that high levels of VP mRNA perinatally allows for the production and storage of VP peptide, which support the extraordinary VP release at birth.

We found very high levels of circulating copeptin in perinatal mice that dropped by 3 h postnatal in vaginally-delivered pups, and more precipitously in pups delivered by C-section. This is consistent with clinical reports that the copeptin/VP surge drops rapidly after birth and is dampened in babies born by C-sections^13–15^. VP mRNA levels were similar at 3 h postpartum in C-section and vaginally-delivered pups, suggesting that release, not production, of VP is sensitive to birth mode. However, neural activation (c-Fos+ cells overall and of VP neurons) was also similar between birth modes. While c-Fos labeling (i.e., the number of c-Fos+ cells and the amount of immunoreactivity per cell) correlates with stimulus intensity^52^, differences between groups may not be detectable past a neural activity and/or protein expression threshold. Therefore, it is possible that c-Fos labeling is at ceiling in vaginally-born mice at 3 h, or that production and release of copeptin/VP are similar by birth mode, but that metabolism of the peptides are accelerated following a Cesarean delivery.

Activation of VP neurons could occur in response to, or in preparation for, the stressors that newborn mammals experience, including immune challenge and hypoxia^53,54^. Birth is an inflammatory event, with the fetus exposed to maternal cytokines during late gestation and to antigens from the plethora of microbes encountered at parturition^55–60^. The PVN coordinates the central response to peripheral infection or inflammation, and shows c-Fos immunoreactivity within 3 h of a peripheral immune challenge^61–63^. Therefore, it would be interesting to determine if neural activation in the newborn is altered by the suppression of immune/inflammatory signals or in sterile (germ-free) mice.

Newborn mammals, including humans, almost invariably experience hypoxia in association with both a vaginal and C-section birth^15,54,64,65^. Hypoxia increases peripheral and central copeptin/VP release in rats, and umbilical cord copeptin is higher at birth in human newborns suffering from birth asphyxia^65,66^. Thus, the hypoxia associated with birth may trigger activation of VP neurons and consequent VP release. Because hypoxia is greater in premature than in full-term infants^67,68^, this may explain why we observed even greater neural activation 3 h after an early birth than after an on-time delivery in mice.

VP released by magnocellular SON and PVN neurons controls blood osmolality and electrolyte balance^33^. In addition, VP neurons of the SCN play a crucial role in osmolality and thirst in mice via central projections^69,70^. Despite high copeptin concentrations in E18.5 fetuses, osmolality was within the normal range. This was expected, due to maternal control of osmolality via the shared maternal–fetal blood supply^71–73^. However, 1-3 h postnatal, we found an acute decrease in plasma osmolality in pups born vaginally which, to our knowledge, has not previously been reported. We hypothesize that this results from the abrupt severing of the maternal blood supply in the face of elevated fetal VP. We cannot rule out the possibility that the drop in osmolality is the *cause* of higher neonatal plasma copeptin/VP in vaginally-delivered pups^74^; if so, however, it would be necessary to explain what causes an abrupt osmolality change at birth. The transient decrease in plasma osmolality was attenuated in pups delivered by C-section, which is consistent with the lower copeptin levels measured in C-section delivered mouse pups (current study) and human babies^13–15^.

An acute decrease in plasma osmolality after a vaginal birth could be an important adaptation to conserve water until lactation is established. Human babies delivered via C-section are more likely to suffer from dehydration and/or hypernatremia in the postnatal period than babies delivered vaginally^75–77^. Whether this is related to birth-mode differences in VP release and resultant effects on plasma osmolality is not known, and is an important area for future study.

Copeptin/VP levels in cord blood correlate closely with those in cerebrospinal fluid of human newborns^36,37^, indicating concomitant peripheral and central release. The SCN and PVN are the main contributors of VP in cerebrospinal fluid^38^, and VP is released from the dendrites of magnocellular VP neurons in the rat hypothalamus in an activity-dependent manner^78,79^. Thus, it is likely that birth causes VP release within the mouse brain. We found that cell death in the PVN and neighboring AHA was markedly suppressed in C-section-delivered pups treated intracerebroventricularly with VP at birth, with no effect in the SON and LHb. This suggests that central release of VP may alter the magnitude of neuronal cell death in newborns. The regional differences we observed may reflect differences in the distribution of vasopressin receptors at birth^80^, but could also reflect the fact that following an icv injection, regions adjacent to the ventricles (e.g., the PVN and AHA) may have experienced greater concentrations of VP than those at a distance.

In addition to neurodevelopment, several other functions could be altered by central VP release at birth. For example, VP has been reported to promote neonatal analgesia^81^ and a recent study demonstrates an inverse correlation between copeptin levels in umbilical cord blood of newborns and sensitivity to a mildly painful stimulus^82^. Central VP may also affect thermoregulation, social behavior, and other functions^83,84^.

The ubiquitous nature of birth may have led us to overlook its importance as a developmental signal. Birth is a dramatic event for placental mammals, and it would be surprising if the fetal/newborn brain was not equipped with mechanisms to face the upcoming challenges. Collectively, our data suggest that birth triggers a neuroendocrine response that may underlie elevated peripheral and central VP, and cause fluid retention and neuroprotection in the newborn. The finding that some of these processes are altered by birth mode has implications for current obstetric practices in which non-vaginal delivery has become increasingly common across the world.

## Materials and methods

### Animal procedures

#### Animals

Adult C57BL/6J mice were purchased from The Jackson Laboratory (Bar Harbor, ME) or obtained from our colony. Adult Wistar rats were purchased from The Charles River Laboratory (Wilmington, MA). A total of 204 mice and 35 rats were used for all reported experiments. Mice and rats were kept in a 12 h:12 h light dark cycle with ad libitum access to water and food. All procedures were approved by the Georgia State University Institutional Animal Care and Use Committee (protocol #A18062) and performed in accordance with ARRIVE and the National Institutes of Health Guide for the Care and Use of Laboratory Animals. CRH reporter mice *(CRH-ires-Cre x Ai14D)* were generated by breeding C57BL/6J mice expressing Cre recombinase under the control of endogenous promoter/enhancer elements of the CRH locus (#012704, Jackson Laboratory) with mice containing a conditionally activatable fluorescent locus [Gt(ROSA)26Sor] that included transcriptional stop cassettes flanked by loxP sequences (#012704, Jackson Laboratory). Offspring express tdTomato specifically in CRH-producing cells, as has been previously validated for this cross^85^*.*

#### Timed pregnancies and delivery mode

We established timed-pregnancies by housing breeding pairs together within 2 h of lights off. Males were removed the next morning, 1–2 h after lights on, and that day was designated embryonic day (E) 0. Starting at E18.5, expectant females were checked hourly around the clock for any signs of labor until vaginal delivery. Brains of male and female pups were collected at 1 h, 3 h, or one day postpartum (P1). We also collected brains of pups delivered via C-section at E18 and E19. Because c-Fos may be expressed in a circadian fashion^86^, C-sections on E19 were yoked to vaginal births as reported previously^29^. Immediately after one female delivered her first pup vaginally, another dam not showing signs of labor was selected for C-section delivery to match time of day and total gestation length. In addition, all brains collected on P1 were harvested at 27 h after birth (i.e., at the same time of day as the 3 h collections). Dams were euthanized using 2% CO_2_ followed by rapid decapitation. An aseptic abdominal incision was made to expose the uterine horns, and the fetuses were removed one by one from their gestational sacs. The brains of C-section-delivered pups were collected immediately after C-sections (0 h), or pups were kept on a heating pad at 32 °C until brain collection at 3 h postnatal on E18 or E19. Offspring from vaginal births that were yoked to C-section births were also kept on a heating pad at 32 °C until brain collection at 3 h postnatal on E19.

### Tissue processing

#### Neural activation experiments

Brains were immersion-fixed in 4% paraformaldehyde for 24 h, transferred into 30% sucrose solution, and coronally frozen-sectioned into four, 40 µm series. Sections were then processed for immunohistochemical detection of c-Fos, or immunofluorescent double labeling of c-Fos and VP, or c-Fos and OT. Unless otherwise noted, all rinses and incubations were performed in 0.01 M phosphate-buffered saline (PBS, pH: 7.4).

#### c-Fos immunohistochemistry

Free-floating sections were rinsed and submerged in concentrated blocking solution [20% normal goat serum (NGS), 0.4% Triton X-100, 1% H2O2] for 1 h. Sections were then incubated overnight in primary antibody solution [rabbit anti-c-Fos 1:5,000 (SC-52, Santa Cruz Biotechnology, Dallas, TX), 2% NGS, 0.4% Triton X-100)] at room temperature. The next day, sections were washed and incubated in secondary antibody solution [biotinylated goat anti-rabbit 1:500 (Vector Laboratories, Burlingame, CA), 2% NGS, 0.3% Triton X-100] for 1 h. Next, sections were washed and then incubated in an avidin–biotin complex solution [1:500 (Vectastain Elite ABC Kit; Vector Laboratories)] for 1 h. Following rinses in sodium acetate buffer, sections were incubated for 30 min in diaminobenzidine-nickel solution, rinsed, mounted onto slides, counterstained with neutral red, and coverslipped.

#### c-Fos and VP or OT immunofluorescent double labeling

Alternate free-floating sections were rinsed and incubated in 0.05 M sodium citrate at 70 °C for 1 h. Sections were then rinsed, placed in 0.1 M glycine for 30 min, rinsed, incubated in a concentrated blocking solution (20% NGS, 0.4% Triton X-100, 3% H_2_O_2_) for 1 h, and then incubated overnight at 4 °C in primary antibody solution against c-Fos [rabbit anti-c-Fos 1:100 (SC-52, Santa Cruz Biotechnology, or 9F6, Cell Signaling Technology, Beverly, MA), 2% NGS, 0.4% Triton X-100]. The next day, sections were washed in a dilute blocking solution (2% NGS, 0.4% Triton X-100), and incubated for 2 h at 4 °C in secondary antibody solution [goat anti-rabbit Alexa 594 1:250 (A11037, Vector Laboratories) for wildtype mice, or goat anti-rabbit Alexa 488 1:500 (A11073, Vector Laboratories) for CRH-tdTomato reporter mice, 2% NGS, 0.4% Triton X-100]. Sections were washed in dilute blocking solution, and then incubated overnight at 4 °C in primary antibody solution against VP or OT [rabbit anti-vasopressin 1:1,000 (PC234L, EMD Millipore, Burlington, MA), or rabbit anti-oxytocin 1:1000 (T-4084, Peninsula Laboratories International Inc., San Carlos, CA), 2% NGS, 0.4% Triton X-100). The next day, sections were washed in a dilute blocking solution and incubated in secondary antibody solution for 2 h at 4 °C [goat anti-rabbit Alexa 488 1:500 (A11073, Vector Laboratories) for wild type mice, or goat anti-rabbit Alexa 350 1:200 (A11046, Vector Laboratories) for CRH-tdTomato reporter mice, 2% NGS, 0.4% Triton X-100)]. To reduce background auto-fluorescence, sections were washed and incubated in 100 mM cupric sulfate in 50 mM ammonium acetate for 1.5 h at room temperature. Finally, sections were rinsed in 1X Tris-Buffered Saline and mounted onto microscope slides using Fluoromount medium (Sigma Aldrich, St. Louis, MO).

#### Cell death experiment

Brains were collected at 3 h postnatal, fixed in 5% acrolein for 24 h, transferred into 30% sucrose solution, and coronally frozen-sectioned into four 40 µm series. Two series of sections were processed for immunohistochemical detection of activated caspase-3 as reported previously^29^.

### Image capture and analysis

#### Light microscopy

Brain regions were identified using well established anatomical landmarks^87^. Regions of interest were outlined bilaterally using Stereo Investigator software (MBF Bioscience Inc., Williston, VT). The overall cross-sectional area and the number of c-Fos+ or AC3 + cells within each trace were recorded. The volume of each brain region was determined by summing the areas of all sections and multiplying by section thickness. c-Fos+ and AC3 + cell density was calculated by dividing the total number of positive cells per animal by the volume of the brain region in mm^3^.

#### Fluorescent microscopy

Fluorescently labeled sections were magnified to 100X and imaged using structured illumination microscopy (Apotome.2, Zeiss, Oberkochen, Germany) and Stereo Investigator software (MBF Bioscience Inc.). The Apotome system eliminates the out-of-focus blur from other focal planes computationally, and achieves spatial resolution comparable to that of standard confocal microscopy^88^. Z-stacks of images in areas of interest were captured to quantify c-Fos/VP, c-Fos/OT, or c-Fos/CRH double-labeled cells; each z-step was 2 μm with an average of about 12 optical sections per image. We using the Cell Counter plugin of Image J (NIH, Bethesda, MD) to mark each subtype within z-stacks of the areas of interest. All analyses were performed by researchers blind to experimental conditions.

### Mouse copeptin enzyme-linked immunosorbent assay (ELISA)

Copeptin concentrations in perinatal mouse plasma were measured using a commercially-available sandwich ELISA kit (EKC36659, Biomatik, Ontario, Canada), following the manufacturer’s instructions. Trunk blood was collected from male and female offspring at E18.5, immediately after birth (0 h) and at 3 h following a vaginal delivery, or at 0 h and 3 h following a C-section delivery. C-sections were yoked to vaginal deliveries to match the time of day and gestation length, as above. We also collected blood from six pregnant dams [two at E18.5, and four at 0 h post-delivery (two vaginal and two C-section)], as well as three non-pregnant adults (two female) that were euthanized using 2% CO_2_ followed by rapid decapitation.

Blood was collected in EDTA-coated capillary tubes (Fisher Scientific, Waltham, MA) containing protease inhibitors (1X Complete-mini, Roche, Darmstadt, Germany). Samples were then transferred into clean microcentrifuge tubes, spun at 1000×*g* at 4 °C for 15 min, and plasma was collected and stored at − 80 °C until analysis (< 1 month). Because very low volumes could be obtained from perinatal mouse pups, plasma from 2 to 3 pups of the same sex from different litters was pooled to form single samples for analysis. All plasma samples were diluted fourfold in PBS and assayed in duplicate following the manufacturer’s instructions. The plate was read immediately using a multimode plate reader at 450 nm (iMark Microplate Reader; Bio-Rad, Hercules, CA). The intra- and inter-assay coefficients of variability (CV) were ≤ 10%, and the CV cutoff was 20% for all data replicates. The sensitivity of the assay was 4.36 pg/mL. The manufacturer reports no significant cross-reactivity or interference between mouse copeptin and analogues. Curve fit and concentration values were calculated using the Microplate Manager 6 Software.

### Quantitative real-time PCR (qRT-PCR)

Brains of male and female offspring were collected via rapid decapitation on E16.5, E18.5, E19, and at 3 h postnatal following a vaginal or C-section delivery. C-sections and vaginal deliveries were yoked, as above. We also collected the brains of vaginally born offspring at P23 after euthanasia with 2% CO_2_ followed by rapid decapitation. Brains were immediately frozen and stored at − 80 °C. PVN punches of 0.8 mm inner diameter were taken in a cryostat and homogenized in TRIzol (Invitrogen, Carlsbad, CA). RNA was precipitated and reverse transcription was performed with a Superscript IV kit (Invitrogen; 300 ng of RNA in 20 µL total volume) in a thermal cycler (Applied Biosystems Inc., Foster City, CA). Real-time PCR was performed in the LightCycler 96 System (Roche, Mannheim, Germany) using FastStart Essential DNA Green Master Kit (Roche), according to the manufacturer’s instructions. Validated primers were used to amplify VP and GAPDH mRNA (QT00249389 and QT01658692, respectively; Qiagen Inc., Valencia, CA), and average quantitative cycle (Cq) values were used to quantify expression of VP relative to the control gene (GAPDH) for each animal using the 2^-ΔΔCq^ method. Values for E16.5 were used as the calibrator group.

### Plasma osmolality measurements

Trunk blood of male and female offspring was collected at E18.5 or E19, and postnatally at 1 h, 3 h, or P1 following a vaginal delivery or at 3 h following a C-section delivery. Blood was collected in heparin-coated capillary tubes (Fisher Scientific), transferred into clean microcentrifuge tubes, and centrifuged at 1680×*g* at 4 °C for 5 min. Plasma was collected and stored at -80 °C until analysis. Plasma samples were diluted twofold in nanopure water and osmolality was measured using the Advanced Micro Osmometer (Advanced Instruments, Norwood, MA).

### Intracerebroventricular (ICV) injections

We injected 250 ng of VP (V9879, Sigma Aldrich, in 500nL artificial cerebrospinal fluid, Tocris Bioscience, Bristol, UK), or the vehicle alone, into each lateral ventricle of cryoanesthetized pups within 30 min of C-section delivery at E19. The dose was based on previous reports in adult mice and infant rats^89,90^, and ICV injections were performed as described previously^91^. Briefly, a 30-gauge needle attached to a 5 µl Hamilton syringe was lowered 2 mm below the skull, at approximately 1 mm rostral to lambda and 1 mm lateral to the sagittal suture. Solutions were injected at a rate of 33 nL/s into the brain of each pup using a Micro4 micro syringe pump (World Precision Instruments, Sarasota, FL). Pups were kept on a heating pad at 32 °C until brain collection 3 h later.

### Statistical analyses

Sex was included as a factor for all analyses but had an effect on only one measure. Therefore, results are reported for both sexes combined, unless noted otherwise. Parametric statistics (one- and two-way ANOVAs or t-tests) were used for datasets that passed tests of normality (Shapiro–Wilk) and homogeneity of variance (Bartlett’s test). If normality was not met, data were transformed (square root or logarithmic), and if normality was still not met, non-parametric statistics were used (Kruskal–Wallis, Mann–Whitney U). A mixed-effect ANOVA was used to examine effects of age (between-subjects factor) and neural phenotype (within-subjects factor) on c-Fos labeling. When applicable, ANOVAs were followed by Fisher’s least significant difference post hoc tests, whereas Kruskal–Wallis tests were followed by Dunn’s post hoc tests. All analyses were performed using GraphPad Prism.

## Supplementary Information

Supplementary Information.
