# Supplementary material for: Birth elicits a conserved neuroendocrine response with implications for perinatal osmoregulation and neuronal cell death
Source: Sci Rep. 2021 Jan 27;11:2335. doi: 10.1038/s41598-021-81511-1 (PMC7840942; doi:10.1038/s41598-021-81511-1)
Supplement: Supplementary file 1 — Supplementary Information. [file 41598_2021_81511_MOESM1_ESM.pdf]

Birth elicits a conserved neuroendocrine response with implications for perinatal osmoregulation and neuronal cell death

Yarely C. Hoffiz[1], Alexandra Castillo-Ruiz[1], Megan A. L. Hall[1], Taylor A. Hite[1], Jennifer M. Gray[1], Carla D. Cisternas[1,2], Laura R. Cortes[1], Andrew J. Jacobs[1] and Nancy G. Forger[1,*]

Supplementary Materials

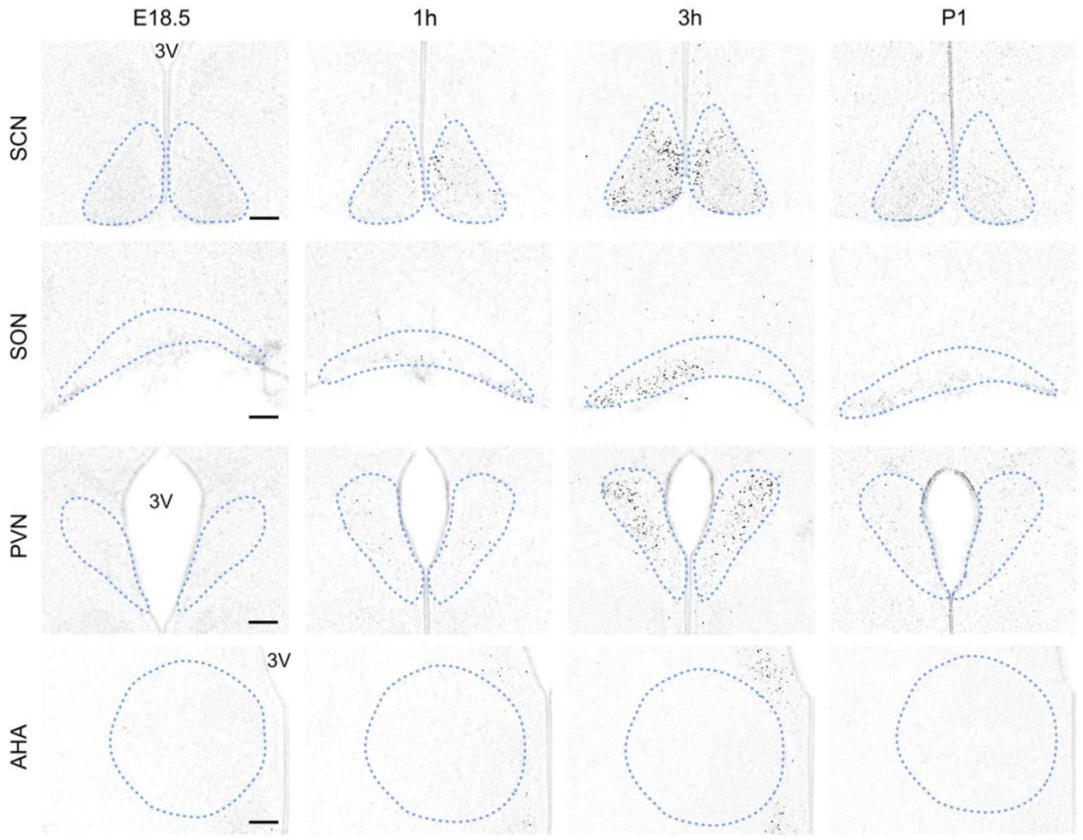

Fig. S1. c-Fos immunoreactivity in the SCN, SON, PVN, and AHA one day before birth (E18.5), and at 1h, 3h, or P1. Photomicrographs show very few c-Fos+ cells at E18.5. Scattered cells within the SCN, SON, and PVN were c-Fos+ at 1h postnatal, and c-Fos+ cell number increased markedly at 3h. At P1, c-Fos labeling was again low. c-Fos+ cell number in neighboring AHA was extremely low at all time points. 3V, third ventricle. Scale bar: 100 µm.

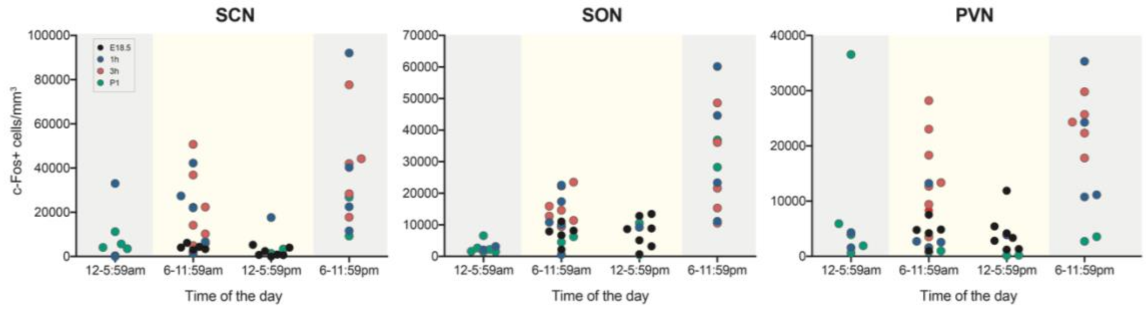

Fig. S2. Neuronal activation plotted as a function of the time of day when brains were collected. Circles depict individual data points for animals collected prenatally at E18.5 (black circles) or postnatally at 1h (blue), 3h (red), or 1 day (P1, green) after delivery. Regardless of time of day of brain collection, c-Fos+ cell density tended to be low in fetuses at E18.5 and high in neonates at 3h postnatal. Shading indicates light condition at the time of collection: lights on - yellow; lights off - gray.

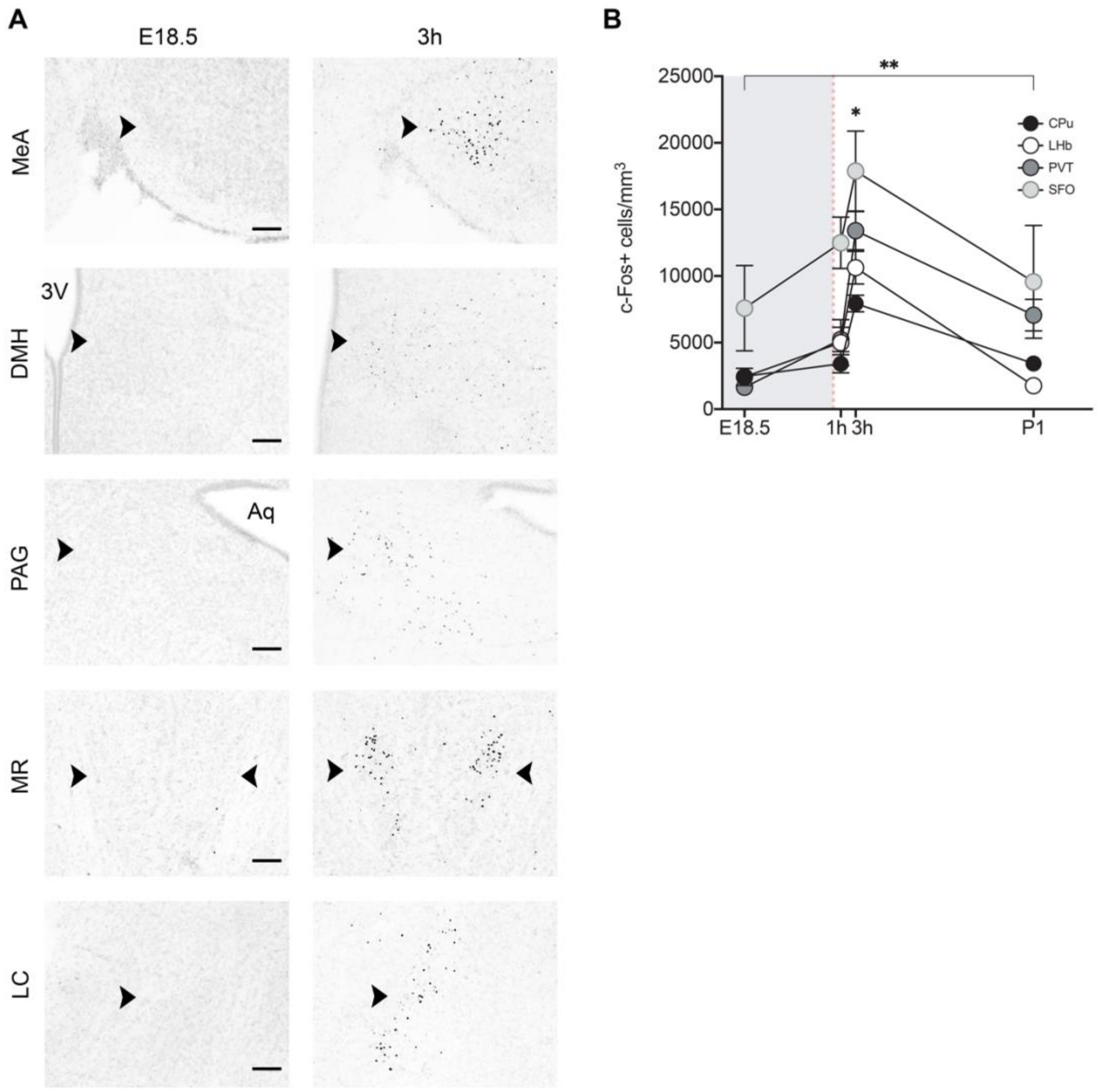

Fig. S3. Increased c-Fos immunoreactivity at 3h postnatal was also observed in other brain areas. (**A**) Photomicrographs show increased c-Fos+ cell number at 3h compared to E18.5 in the medial amygdala (MeA), dorsomedial hypothalamus (DMH), periaqueductal gray (PAG), median raphe (MR, shown bilaterally) and locus coeruleus (LC) as highlighted by the arrowheads. 3V, third ventricle; Aq, cerebral aqueduct. Scale bar: 100 µm. (**B**) Quantification of c-Fos+ cell density showed that c-Fos immunoreactivity was also moderately increased in the caudate/putamen (CPu), paraventricular nucleus of the thalamus (PVT), lateral habenula (LHb), and subfornical organ (SFO) at 3h postnatal compared to E18.5 and P1 (*$P < 0.02$ for all areas). Asterisks over bracket indicate significant main effect of age on c-Fos+ cell density in all brain areas (**$P < 0.007$). Gray shading indicates *in utero* timepoints; red dotted lines indicate the time of birth. Data are mean ± SEM. n = 10-12 animals per group.

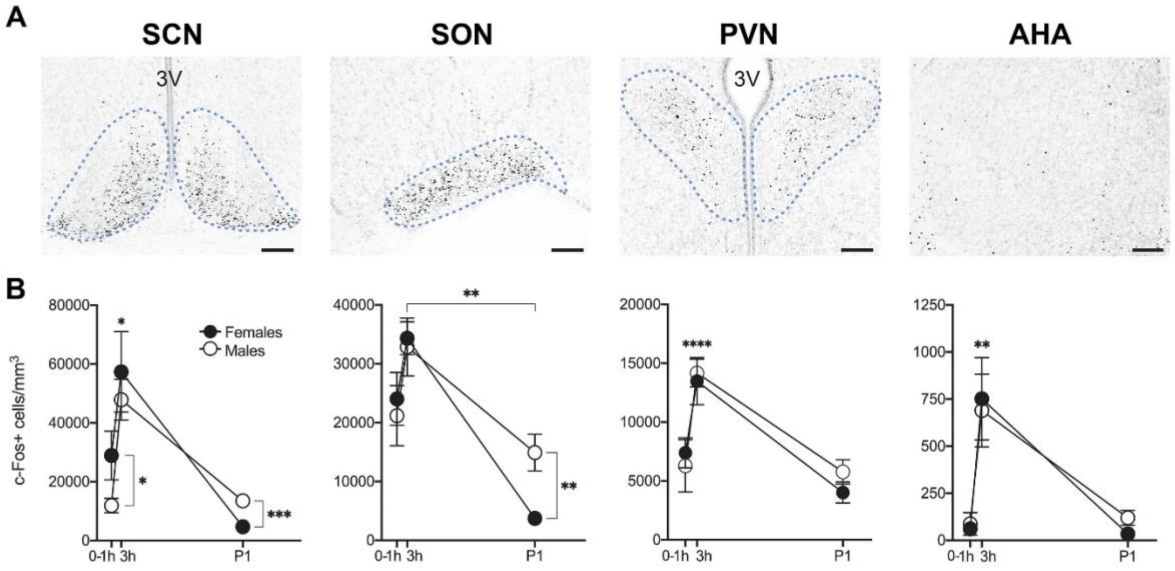

Fig. S4. Birth induces neural activation in a sex-dependent manner in specific hypothalamic regions of rats. (**A**) c-Fos immunoreactivity is elevated in the rat SCN, SON, PVN, and AHA at 3h post-delivery. 3V, third ventricle. Scale bar: 100 µm. (**B**) In the SCN, there was a main effect of age ($F_{2,29} = 37.2$, $P < 0.0001$), with higher c-Fos immunoreactivity at 3h postpartum (3h vs 0-1h and P1, *$P < 0.04$) as well as an age-by-sex interaction ($F_{2,29} = 10.03$, $P = 0.0005$). Males had higher c-Fos expression at P1 (asterisks next to vertical bracket, ***$P = 0.0008$), whereas females had higher c-Fos expression within 1h of birth (asterisk next to vertical bracket, *$P < 0.02$). In the SON, there was a main effect of age ($F_{2,22} = 32.09$, $P < 0.0001$), with higher c-Fos+ cell density at 3h postpartum compared to P1 (**$P < 0.001$); only males had significantly higher c-Fos+ cell density at 3h postpartum compared to 0-1h ($P = 0.01$). There also was an age-by-sex interaction ($F_{2,22} = 6.19$, $P = 0.007$) reflecting that males had higher c-Fos expression at P1 (vertical bracket, **$P = 0.004$). In the PVN and AHA, c-Fos+ cell density was highest at 3h postpartum in the female and male rat (3h vs 1h or one day after birth (P1), **$P < 0.01$, ****$P < 0.0001$; both sexes combined, as c-Fos+ cell density did not differ by sex). c-Fos+ cell density was 20-80 times lower in the AHA. Data are mean ± SEM. n = 6 per group.

Table S1. Total number of cells immunoreactive for each peptide at E18.5, 3h postnatal and P1.

Brain Area	Neuropeptide	Age	Mean cell density (Number of cells/mm^3)	Main effect of age (Post hocs if significant)
SCN	VP	E18.5	11,792 ± 2,561	H_2 = 6.745, *P* < 0.04
		3h	21,925 ±1,782	(E18.5 vs. 3h, *P* < 0.01)
		P1	17,500 ± 3,300	
SON	VP	E18.5	83,340 ± 7,450	n.s.
		3h	81,287 ± 10,922	
		P1	82,702 ± 10,744	
	OT	E18.5	3,399 ± 557	H_2 = 9.04, *P* < 0.0001
		3h	8,475 ± 895	(E18.5 vs. 3h, *P* < 0.005)
		P1	12,153 ± 1,426	(E18.5 vs. P1, *P* < 0.0001)
PVN	VP	E18.5	11,352 ± 1,031	n.s.
		3h	13,856 ± 1,913	
		P1	12,420 ± 2,464	
	OT	E18.5	8,356 ± 815	n.s.
		3h	6,787 ± 889	
		P1	8,461 ± 537	
	CRH	E18.5	16,935 ± 6,029	n.s.
		3h	25,234 ± 6,151	
		P1	27,574 ± 6,229	
